# Social brain responses to natural scene images of social interactions

**DOI:** 10.1093/scan/nsaf057

**Published:** 2025-06-26

**Authors:** Ilona Martynenko, Kami Koldewyn, Paul E Downing

**Affiliations:** Institute of Cognitive Science, University of Osnabrück, Osnabrück, 49069, Germany; Department of Psychology, Bangor University, Bangor, LL572AS, United Kingdom; Department of Psychology, Bangor University, Bangor, LL572AS, United Kingdom

**Keywords:** social interaction, person perception, body perception, functional neuroimaging, occipitotemporal cortex

## Abstract

Recent research reveals that human occipitotemporal ‘social brain’ regions that are selective for images of individual faces and bodies are also sensitive to visual cues of social interaction. Earlier studies mainly contrasted observing dyadic interactions with non-interactive controls, emphasizing the interacting/non-interacting distinction to observers, and lacking the variety seen in natural settings. To address these limitations, we analysed a 7 T fMRI data set in which participants viewed many naturalistic images while performing a memory task. We focused on 182 scenes containing at least two individuals, and used localizers to identify face- and body-selective regions of interest (ROIs). Brain responses to each image were measured, and the depiction of social interaction was rated by independent observers. Control measures were gathered, per image, for the number of people, their surface area and distribution, and their implied animatedness. Linear and generalized additive modelling revealed that social interaction predicted a greater BOLD response in all ROIs, beyond the effects of the control variables. Face- and body-selective regions in both hemispheres showed heightened sensitivity to social interaction in natural scenes, even during an orthogonal task. These findings expand our understanding of ‘social vision’ areas beyond individual person perception to include multi-person social interactions.

## Introduction

Our visual environment is rich with cues that can help navigate social life. The faces, bodies, and movements of other people provide information about their sex, age, ethnicity, health, physical strength, emotion, direction of attention, social status, personality, and goals (Adams 2011). The science of social vision has sought to reveal more about the cognitive processes and representations that make use of these cues and to describe the relevant brain regions and networks (Stolier and Freeman 2016, Pitcher and Ungerleider 2021). In humans, a group of occipitotemporal regions has been identified that respond selectively to faces, bodies, and their movements, and that contribute causally to social perception tasks (Grossman et al. 2005, Pelphrey et al. 2005, Kanwisher and Yovel 2006, Peelen and Downing 2007). Most of this work has examined the brain activity related to tasks performed on depictions of single people, leading to theoretical perspectives on how occipitotemporal regions work together to analyse and synthesize the appearance of other individuals to extract social meaning (Haxby et al. 2000, Taylor and Downing 2011, Grill-Spector and Weiner 2014, Duchaine and Yovel 2015, Pitcher and Ungerleider 2021, Puce 2024).

More recently, neuroscience researchers have examined the brain systems engaged by viewing two or more socially interacting individuals (Isik et al. 2017, Quadflieg and Koldewyn 2017, Abassi and Papeo 2020). Observers stand to learn much about other people by watching them interact with each other—e.g. their relative status or mutual attitudes—even when the observer is not involved in the interaction. This work has identified selective activity in the posterior superior temporal sulcus (pSTS) of humans (and macaques; Freiwald 2020) when they view realistic videos or simplified animations of interacting dyads, relative to controls such as visually-matched but non-interacting pairs (e.g. Landsiedel et al. 2022, McMahon et al. 2023).

Selective responses to images or animations of dyadic social interactions are also found in the human extrastriate body area (EBA; Downing et al. 2001). The EBA responds strongly and selectively to images of individual bodies and body parts in a range of formats, relative to other kinds of non-body objects including faces (Downing and Peelen 2011). Neurostimulation and neuropsychological evidence (Downing and Peelen 2016) establishes a causal role in person detection (van Koningsbruggen et al. 2013) and in shape/posture discrimination (Urgesi et al. 2004, Moro et al. 2008, Pitcher et al. 2009). The functions of this region, alongside a ventral region with a similar functional profile, the fusiform body area (FBA; Peelen and Downing 2005, Schwarzlose et al. 2005), have typically been interpreted in terms of single-person perception—such as in the representation of body posture, identity, or contributing to one’s own motor movements (reviewed in Downing and Peelen 2011).

More recent research points to a more complex role for EBA. Multivoxel pattern analyses show that EBA activity contains latent information about different kinds of observed dyadic interactions presented in videos (Walbrin and Koldewyn 2019). Also, facing static dyads elicit more EBA activity (especially in the left hemisphere) than non-facing pairs, a distinction that is reduced by in-plane inversion (Abassi and Papeo 2020). This inversion effect mirrors a behavioural deficit for making judgements about inverted relative to upright dyads (Abassi and Papeo 2022). The activity of left hemisphere EBA is causally involved in producing this behavioural ‘two-body inversion effect’ (Gandolfo et al. 2024). These results suggest that EBA (and particularly left EBA) represents not only individual bodies and body parts, but is also sensitive to one of the perceptual cues signalling that a social interaction is taking place.

The aim of the current study was to investigate the responses of EBA and other face- and body-selective regions to static images of social interactions, with three main advances on previous work. First, we used a high-quality open science data set comprising neuroimaging and behavioural measures from eight participants who viewed thousands of unique natural scene images while being scanned with high field strength fMRI (Allen et al. 2022). This provided the sensitivity to measure item-level differences in the responses to scene images as a function of their social interaction content. Second, unlike previous studies that contrasted facing vs non-facing dyads, we measured the responses to images that indicate social interaction in richer ways. Finally, the images that participants viewed varied in many ways, including location, presence or absence of people, viewpoint, kinds of objects visible, and so on.

We assessed the responses of left and right EBA, FBA, and fusiform face area (FFA) to images as a function of their social interaction content. Because realistic images include confounds, we also measured, for the same images: the number of people in the image and their left–right distribution; the surface area of the images covered by people; and the extent to which depicted people are shown in dynamic postures that imply motion. In this way, we could assess how social interactions, displayed in a range of ways in naturalistic scene images, modulate the activity of key ‘social brain’ regions, above and beyond the influence of other confounding variables.

There is no single objective definition of what constitutes a ‘social interaction,’ and a given scene may imply a social interaction with different people to different degrees. Multiple image cues, alone or in combination, can indicate the presence of a social interaction. These include facing direction, proximity, mutual eye gaze, touch, and joint attention to an object (Quadflieg and Koldewyn 2017, McMahon and Isik 2023). These considerations informed our ‘bottom-up’ approach: we asked naive external raters to judge scenes on the extent to which they depict a social interaction, without imposing an experimenter-determined definition, and without specifying ‘a priori’ what kinds of cues the raters should consider. While there is a risk here that we do not measure exactly what we think we are measuring—because we allow our participants to interpret the construct—in this case we feel the risk is managed, because ‘social interaction’ is not an esoteric concept but a part of normal everyday life.

That approach is also aligned with our method of testing brain responses to a range of unselected natural scenes, rather than lab-controlled stimuli. We embrace the complexity of realistic images, accepting that we do not know about all of the underlying cues, but avoiding the limitations of an analytical approach. We suggest that this bottom-up, data-driven design complements, without replacing, more traditional carefully controlled designs aimed at isolating specific image properties. One particular advantage of avoiding such a design is that we avoid highlighting the manipulated dimensions to participants’ attention, which could artificially inflate their impact on judgments.

## Materials and methods

These procedures were approved by the Bangor University School of Psychology and Sport Science Ethics Committee.

### Stimuli and materials

Our initial search for suitable datasets included several open resources. We considered and excluded the Natural Object Dataset (Gong et al. 2023). This large dataset includes 3 T fMRI data from participants who collectively viewed many images, as well as functional localizers for category-selective regions. However, most of the images were of single isolated objects, and only a very small proportion included multiple people in scenarios that may be interpreted as interactions. Similarly, the ‘THINGS’ dataset (Hebart et al. 2023) focuses on images of individual objects rather than social scenes. Finally, the BOLD5000 dataset (Chang et al. 2019) includes neuroimaging data from participants who viewed many natural scene images, but did not include localizers for face or body selective regions.

Accordingly, we used fMRI data from the Natural Scenes Dataset (Allen et al. 2022). The study adopted a rapid event-related design in which participants viewed thousands of unique images. From the total set of images, 907 were viewed by each of the eight participants at least once, and 515 were seen by everyone three times. During fMRI scanning, a continuous recognition task was used, in which participants reported whether the image being shown had already been seen. The dataset includes estimates of the BOLD response to every trial in each voxel. For each participant, a functional localizer task was conducted with five main ‘domains’ (faces, characters, bodies, objects, and places), and within each domain two more specific categories (body/limb for bodies; child/adult for faces).

We first identified the 907 images seen by all participants. To further limit the set to potentially relevant materials, only images containing at least two people were selected for the study, leaving 189 images. In the work of Allen et al. (2022) the images (which came from the COCO dataset; Lin et al. 2014) were cropped to squares of 8.4° × 8.4°. Here, we used the full original COCO images without any cropping.

### Characterization of images

**Figure 1.**
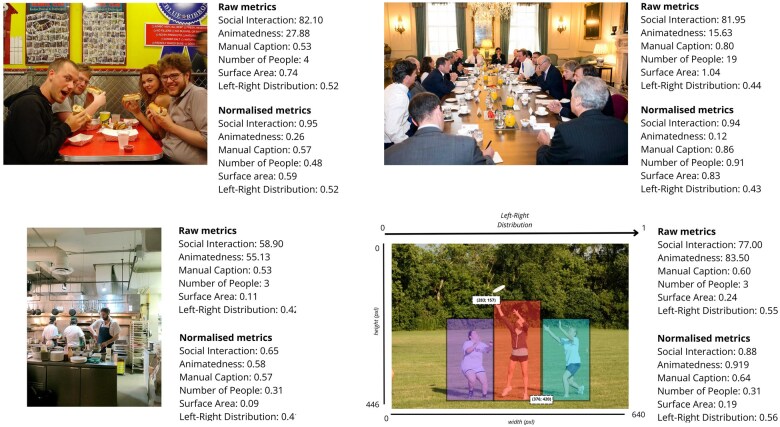
Four sample images (Lin et al. 2014) along with derived subjective and objective metrics of social interaction and other control variables. For each image, we provide metrics as mean raw measures and as normalized measures. The bottom-right image illustrates the application of automated person detection and localization, which was used to compute surface area and left-right distribution metrics.

In Fig. 1, we present four of the images that we used, along with subjective and objective measures for each regarding social interaction, animatedness, number of people, and the spatial distribution and surface area of people in the image. These measures are described in turn below.

#### Social interaction

We collected online ratings from 25 naïve participants (sample size set arbitrarily) about the level of social interaction depicted in the images selected for this study. All participants provided their informed consent to participate. With Gorilla Experiment Builder (www.gorilla.sc; Anwyl-Irvine et al. 2020), participants rated 189 images presented one at a time at the centre of their screen. Participants were instructed to ‘provide a rating of the extent to which [each image] shows ‘social interactions among two or more people.’ Ratings were provided by adjusting on on-screen slider, with anchor points labelled ‘No interaction’ at the left, and ‘Strong interaction’ at the right. There was no time limit to respond. The slider started at the centre point in each trial. Slider responses were initially re-coded on a linear scale from 0 to 100. In pre­processing, individual responses were excluded if they were deemed to be too rapid (<2600 ms). Further, 10% of participants whose responses deviated by more than 2 SD from the group average across all images were considered as outliers and their data was removed, leaving 20 datasets. Finally, all participant ratings were scaled to range from 0 to 1. We computed intra-class correlation (ICC3k) amongst the remaining 20 raters on this measure (using the R ‘psych’ package; Revelle 2025) for average fixed raters, which resulted in an agreement score of 0.96, indicating strong agreement.

#### Animatedness

A similar approach was adopted to derive subjective measures of the extent to which each image depicted dynamic human movements. A new group of online 29 participants were instructed to judge ‘how much the people in the scene are performing dynamic, active movements,’ The endpoints of the on-screen slider read ‘No dynamic, active movements’ on the left, and ‘Strongly dynamic, active movements’ on the right. Outlier elimination was applied as described above, leaving data from 25 participants.

#### Manual captions

We also characterized the selected scenes by examining their captions in the COCO database (Lin et al. 2014). Each of the selected images was annotated with five caption sentences written by five people. With WordNet (Fellbaum 1998, Wallace 2007, Feinerer and Hornik 2023) we analysed these captions, to focus on the presence of words indicating plurality, person nouns, and interaction verbs. The intention was to use these text responses as an additional indicator of social interaction depicted by the images. Specifically, we counted the number of appearances in each image caption of person nouns in the plural form (e.g. ‘girls’ and ‘boys’), the plurality of people (e.g. ‘three’ or ‘a group of’), and interaction verbs (e.g. ‘meet’). The verbs were chosen from VerbNet (Schuler 2005). Instances were summed up and divided by the number of sentences and the number of classes (to avoid penalizing a sentence for lacking one or more class types in an image caption). For example, the sentence ‘A group of girls meeting in the yard’ would get the highest score of one, whereas ‘A group of girls standing’ has a lower score of 0.66 to indicate the lack of obvious interaction. Afterwards, the min-max algorithm was applied over the image set, resulting in a coefficient of 0–1 per image.

#### Number of people

We measured the number of people depicted per image in two ways. First, subjective counts were collected for each image from one researcher (IM) and one other independent rater who was naïve to the experimental aims. In some images, this judgement was not trivial, for example where a large group of people was shown in the background of the image. With the ‘irr’ package in RStudio (Gamer et al. 2019) we computed Cohen’s Kappa coefficient (Cohen 1960) over the two raters, which was 0.559. Second, we obtained an objective person count with the ‘You Only Look Once’ (YOLO) algorithm. YOLO applies a single-pass algorithm to detect, localize, and categorize objects in images (including people). Each image was passed to YOLO version 3 (Redmon and Farhadi 2018) and instances of ‘person’ were counted. Fleiss’ Kappa (Fleiss 1971) among the three raters (two human raters and YOLO v3) was 0.467, thus indicating medium reliability of information regarding the number of people in an image. At this point, seven images out of the originally chosen 189 image set were identified as depicting only one person, and hence these were excluded from further analyses, leaving 182 images total. Each image was described by an average number of people depicted, computed over the subjective and objective ratings. These average number-of-persons ratings were scaled with the arctan function, to account for the diminishing significance of additional people in larger groups (e.g. adding one to a dyad versus adding one to a group of 10). Finally, to scale the data in alignment with the other measures, the transformed mean person counts were scaled to a range of 0–1.

#### Surface area

We used the R ‘magick’ package to get information about each image, and the person detections and boundaries from YOLO. With these, we calculated the surface areas of each person detected by YOLO per image and then summed those areas for each picture. The summed area was then divided by the total area of the image for each photo, to derive the proportion of the image surface area occupied by people. Those proportions were then normalized to a 0–1 scale using the min-max algorithm.

#### Left–right distribution of people

Like much of the visual system, body- and face-selective extrastriate regions tend to show a contralateral bias (Hemond et al. 2007, Chan et al. 2010, Silson et al. 2022, Herald et al. 2023). Here we used person detections from the YOLO algorithm to quantify the distribution of people in a given image. For each image, we calculated the non-normalized centre coordinates for each person. Then, each person in an image was given a numerical weight by comparing the surface area of that person relative to the sum of the areas of all persons. Finally, to index the person distribution over an image, we multiplied the weights of each person with their relative left–right position in the image and summed these values. The result is an index for each image ranging from 0 to 1, such that values closer to zero represent a balance of people on the left side of the image, and values closer to one indicate a balance of people towards the right.

### fMRI data

**Figure 2.**
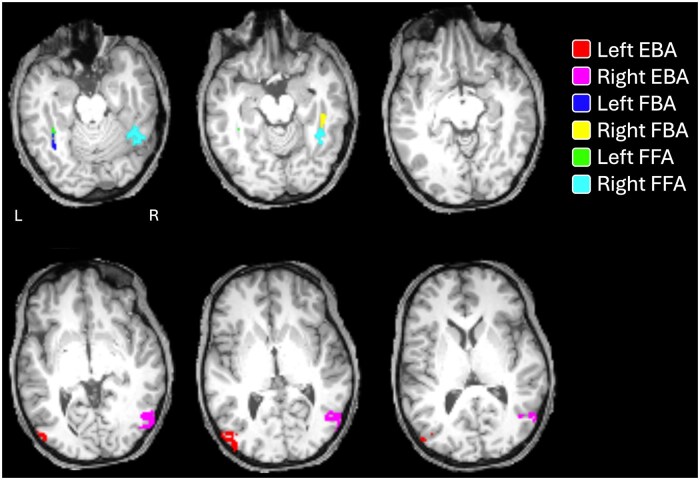
Illustration of regions of interest from one participant. ROIs are depicted as binary masks overlaid on T1 anatomical image from that participant, in native coordinate space. Abbreviations: EBA = extrastriate body area; FBA = fusiform body area; FFA = fusiform face area.

The main outcome measure for this study was the BOLD fMRI response to each selected image, measured in the left and right hemisphere EBA, FFA, and FBA of each participant (see Fig. 2 for an example). All analyses of brain data were conducted in participant-native space. We used the betas from the NSD-provided data from their main experiment, in the native 1.8 mm resolution, which was acquired at a TR of 1.333 s. Our analyses used the per-trial beta images that were fitted with a ‘library-of-HRFs approach,’ GLM denoizing, and ridge regression [these are labelled in Allen et al. 2022, as ‘Beta version 3 (b3)’].

For each participant, each regions of interest (ROI) was identified in each hemisphere from the provided *t* map images (one *t* value per voxel; e.g. ‘floc_bodiestval.nii’) from the functional localizer data, which were in the same space as the main experiment beta maps. The design of the functional localizer experiment was adapted from Stigliani et al. (2015). Briefly, it comprised blocks of images from several different ‘domains’ (characters, bodies, faces, places, and objects), each comprising two sub-categories. Body-­selective regions were identified with a contrast of bodies (including both sub-categories) versus all other categories, and likewise face-selective regions with a contrast of faces versus all others. For each ROI, a local peak voxel consistent with previous reports of these regions was identified with SPM12 (Penny et al. 2011). Next, voxels within a sphere (radius 10 mm) of that peak were included in the ROI if they reached *t* > 5.0 in the localizer dataset. Voxels were allowed to contribute to more than one ROI. (For participant 1, we used a threshold of *t* > 4.0 to identify the left FFA).

Using SPM and the MarsBaR package (Brett et al. 2002) these ROI masks were used to retrieve the beta values from each trial in which images of interest were presented. The beta values were averaged over the voxels within each ROI, for each image. These averages were initially computed separately for each participant and then averaged over participants. These values were submitted as the Y variables in the regression analyses reported below.

## Results

Data were analysed with R version 4.1.1 (R Core Team 2021) in the RStudio environment (RStudio Team 2021) with the following packages: ‘irr,’ ‘gam,’ ‘GGally,’ ‘ggplot2,’ ‘itsadug,’ ‘magick,’ ‘mgcv,’ ‘ppcor,’ ‘tidyverse,’ and ‘WordNet’ (Fellbaum 1998, Wallace 2007, Kim 2015, Wickham 2016, Wood 2017, Gamer et al. 2019, Wickham et al. 2019, Schloerke et al. 2021, van Rij et al. 2022, Feinerer and Hornik 2023, Hastie 2023, Ooms 2023).

### Descriptive statistics

**Figure 3.**
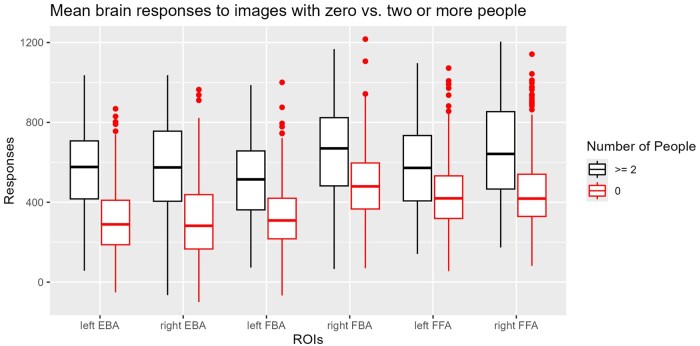
fMRI response in each region of interest to those images seen by all participants, as a function of whether those images contained two or more people (left side for each ROI, in black) or no people (right side for each ROI, in red). Abbreviations: EBA = extrastriate body area; FBA = fusiform body area; FFA = fusiform face area. Horizontal bars indicate the median response. Box boundaries reflect quartile boundaries, whiskers include the range within 1.5 × the inter-quartile range, and points represent individual outlier values.

**Figure 4.**
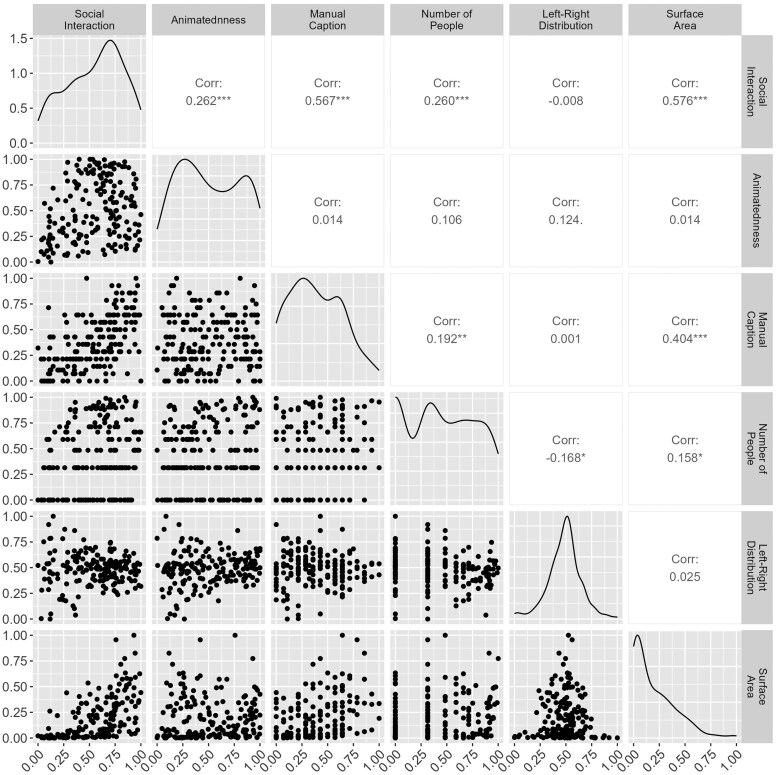
Pairwise correlations between predictor variables (upper triangle), density plots of each of those variables (main diagonal: x-axis is value range, y-axis is density from the probability density function), and the joint distributions of each pair of predictors (lower triangle).

Figure 3 illustrates the mean response in each region of interest to the images that depicted at least two people, and that were seen by all participants, as compared to the remaining commonly seen images that did not depict people. An overview of the distributions of values in our derived measures, and their correlations with each other, is provided in Fig. 4. Note, for example, that the distribution of ‘social interaction’ ratings is negatively skewed, such that our selected images on average tend to have a relatively high level of social interaction depicted. In general, the correlations between measures were modest (all below 0.6). There was a significant correlation between the ratings of social interaction that we collected, and the corpus-derived indices of interaction terms taken from the COCO labels, *r*(362) = .57, *P* < .001. The correlation between the number of depicted people and the social interaction ratings was significantly positive, *r*(362) = .26, *P* < .001, as was the correlation between animatedness and social interaction, *r*(362) = .26, *P* < .001. The correlation between interaction ratings and the proportion of image surface area covered by people was positive and significant, *r*(362) = .58, *P* < .001. These correlations confirm the importance of accounting for these confounding variables, although they are not so high as to prevent models from fitting due to multicollinearity.

**Figure 5.**
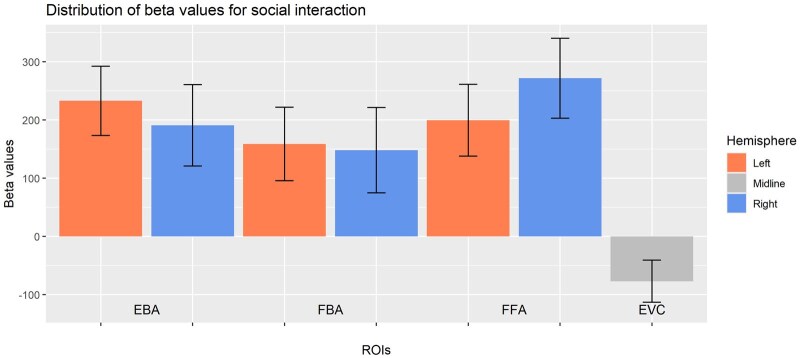
Beta values (in arbitrary units; error bars indicate SE) from linear regression models for the ‘social interaction’ regressor, for each ROI tested.

### Regression models

We used multiple linear regression to model the relationship between the fMRI response of a particular ROI and the predictor variables that describe an image viewed by the participants. The model took the following form:


Brain Response = β0 + β1* Social Interaction Ratings + β2*Number Of People + β3*Left-Right Distribution + β4*Manual Caption + β5*Animatedness Ratings + β6*Surface area + ε

where *β*_0_ is the intercept, *β*_1 − 6_ characterize the change in brain response related to changes in the predictor variables, and ε is the error term. We did not include interaction terms for the sake of interpretability and in the absence of a strong hypothesis requiring them.

To account for the potential non-linearities in the data, we also performed analyses with ‘generalised additive models’ (GAM). This is a type of generalized linear model that uses smoothing parameters to achieve a better fit with the data, including a penalty function towards non-linearity to reduce the likelihood of overfitting. Model fitting was performed with the Restricted Maximum Likelihood (REML) method. In an initial exploration, three models with different types of smoothing parameters—cubic regression spline, thin plate spline, and Gaussian process smoothing terms—were applied to a subset of the predictor variables. These variants produced similar results based on various evaluation metrics, so we report results with only a cubic regression spline smooth term.

#### Results of linear models

Summaries of the linear regression models for EBA, FBA, and FFA are provided in Tables 1–3. All regions responded significantly more strongly to images to the extent that they contained social interactions (as measured by subjective ratings), above and beyond the contributions of the other included variables (see Fig. 5). A *t*-test for hemisphere differences in the EBA’s response to social interactions, motivated by previous findings of a left EBA bias in dyad perception (Gandolfo et al. 2024), was not statistically significant, *t*(175) = .46, *P* = .65.

**Figure 6.**
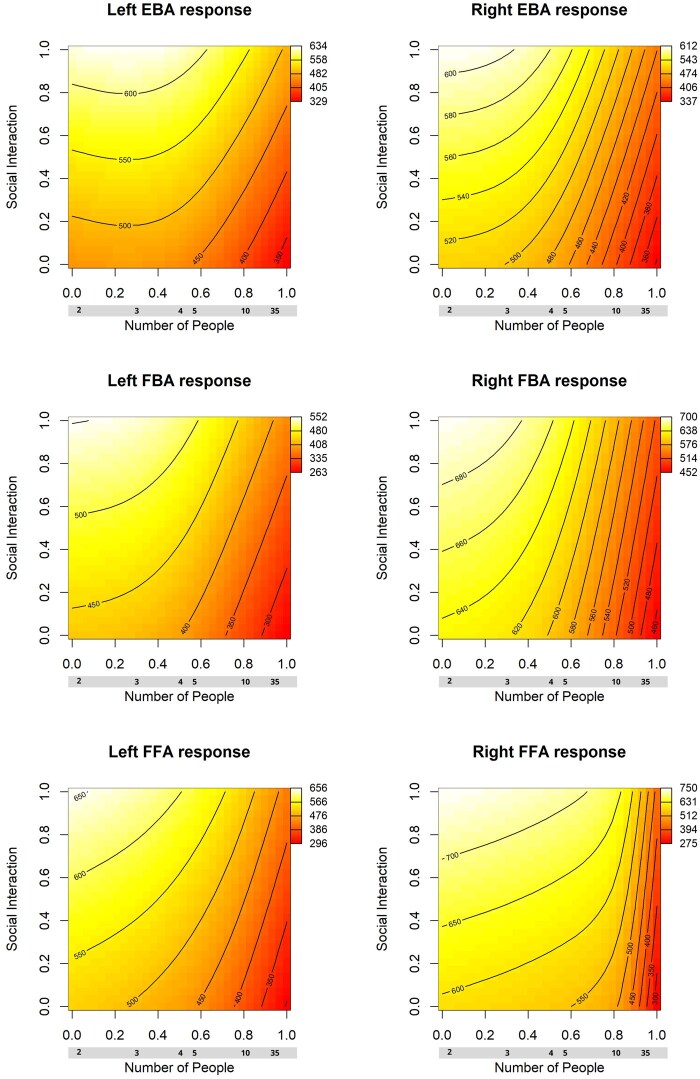
Heat map plots of the fitted relationships between social interaction ratings of images, the number of people they contain, and the mean activity in key brain regions of interest. These are derived from GAM models (see main text). Contour lines reflect estimated curves of constant predicted response as a function of the predictor variables. Note these variables are scaled from 0 to 1 in the modelling. For the number of people variable, the corresponding raw values are shown against the X-axis. The original subjective social interaction ratings were gathered on a continuous scale quantified to a 0–100 range. Note the relative maximum of activity for images with low numbers of people, and high social interaction ratings. Abbreviations: EBA = extrastriate body area; FBA = fusiform body area; FFA = fusiform face area.

**Table 1.** Summary of the linear model results for the analysis of EBA responses.

Left EBA	Estimate	SE	*t*	*P*
(Intercept)	164.17	44.59	3.68	0.000308
Social interaction	232.82	59.34	3.92	0.000125
Animatedness	312.40	39.53	7.90	2.91E-13
Manual caption	24.20	52.47	0.46	0.645243
Number of people	−94.64	34.00	−2.78	0.005969
Left–right distribution	120.00	68.96	1.74	0.083588
Surface area	386.02	64.81	5.96	1.38E-08
Residual standard error: 144.4 on 175 df				
Multiple R-squared: 0.5809, Adjusted R-squared: 0.5665				
F(6, 175) = 40.42, P < 2.2e-16				

Right EBA	Estimate	SE	*t*	*P*
(Intercept)	248.81	52.45	4.74	4.34E-06
Social interaction	190.87	69.81	2.73	0.00689
Animatedness	399.92	46.50	8.60	4.35E-15
Manual caption	−7.95	61.72	−0.13	0.89768
Number of people	−135.63	40.00	−3.39	0.00086
Left–right distribution	−83.35	81.12	−1.03	0.3056
Surface area	523.49	76.23	6.87	1.10E-10
Residual standard error: 169.9 on 175 df				
Multiple R-squared: 0.5588, Adjusted R-squared: 0.5436				
F(6, 175) = 36.93, *P* < 2.2e-16				

**Table 2.** Summary of the linear model results for the analysis of FBA responses.

Left FBA	Estimate	SE	*t*	*P*
(Intercept)	228.55	47.33	4.83	2.98E-06
Social interaction	158.81	62.99	2.52	0.012582
Animatedness	308.69	41.95	7.36	6.96E-12
Manual caption	−9.79	55.69	−0.18	0.86068
Number of people	−143.10	36.09	−3.97	0.000107
Left–right distribution	27.47	73.20	0.38	0.707933
Surface area	409.02	68.79	5.95	1.45E-08
Residual standard error: 153.3 on 175 df				
Multiple R-squared: 0.4975, Adjusted R-squared: 0.4802				
F(6, 175) = 28.87, *P* < 2.2e-16				

Right FBA	Estimate	SE	*t*	*P*
(Intercept)	378.30	54.95	6.88	1.00E-10
Social interaction	148.09	73.14	2.03	0.044401
Animatedness	295.12	48.71	6.06	8.20E-09
Manual caption	5.85	64.67	0.09	0.928017
Number of people	−143.55	41.90	−3.43	0.000764
Left–right distribution	14.81	84.99	0.17	0.861885
Surface area	478.62	79.87	5.99	1.15E-08
Residual standard error: 178 on 175 df				
Multiple R-squared: 0.4453, Adjusted R-squared: 0.4263				
F(6, 175) = 23.42, *P* < 2.2e-16				

**Table 3.** Summary of the linear model results for the analysis of FFA responses.

Left FFA	Estimate	SE	*t*	*P*
(Intercept)	444.02	46.38	9.57	<2E-16
Social interaction	199.71	61.73	3.24	0.00145
Animatedness	−48.90	41.12	−1.19	0.23595
Manual caption	−20.25	54.58	−0.37	0.71105
Number of people	−180.63	35.37	−5.11	8.49E-07
Left–right distribution	14.78	71.74	0.21	0.83698
Surface area	624.94	67.42	9.27	<2E-16
Residual standard error: 150.2 on 175 df				
Multiple R-squared: 0.5467, Adjusted R-squared: 0.5311				
F(6, 175) = 35.17, *P* < 2.2e-16				

Right FFA	Estimate	SE	*t*	*P*
(Intercept)	474.24	51.72	9.17	<2E-16
Social interaction	271.69	68.83	3.95	0.000114
Animatedness	13.07	45.85	0.29	0.775863
Manual caption	−32.62	60.86	−0.54	0.592668
Number of people	−161.84	39.44	−4.10	6.22E-05
Left–right distribution	−70.77	79.99	−0.89	0.377502
Surface area	726.72	75.17	9.67	<2E-16
Residual standard error: 167.5 on 175 df				
Multiple R-squared: 0.5788, Adjusted R-squared: 0.5644				
F(6, 175) = 40.08, *P* < 2.2e-16				

The response of all regions except for FFA was significantly and positively related to subjective measures of animatedness. The relationship between the number of people and brain response was negative in all of the regions tested, likely reflecting that in large groups the visibility of any one person is reduced. In contrast, the responses of all regions were strongly and positively driven by the proportional surface area of the image that was occupied by people. The contribution of manual captions from the COCO database was negligible, perhaps because relevant variance was better captured by the more direct subjective ratings measures.

As an additional control (designed and conducted after the primary results were known) we conducted an identical linear model analysis of the responses in the early visual cortex (EVC) (see Supplementary data). In brief, we used per-participant masks covering V1, V2, and V3 supplied by Allen et al. (2022) to measure per-image responses as described above. We found a significant ‘negative’ relationship between early visual responses and social interaction ratings (see Table 4 and Fig. 5). We conclude that the positive influence of this variable on social brain ROIs cannot be simply ‘inherited’ from earlier visual areas.

**Table 4.** Summary of the linear model results for the analysis of EVC responses.

EVC	Estimate	SE	*t*	*P*
(Intercept)	538.66	27.22	19.79	<2E-16
Social interaction	−77.10	36.23	−2.13	0.0347
Animatedness	−140.90	24.13	−5.84	2.50E-08
Manual caption	46.78	32.03	1.46	0.1460
Number of people	17.15	20.76	0.83	0.4097
Left–right distribution	−37.15	42.10	−0.88	0.3788
Surface area	168.31	39.56	4.25	3.41E-05
Residual standard error: 88.17 on 175 df				
Multiple R-squared: 0.2993, Adjusted R-squared: 0.2753				
F(6, 175) = 12.46, *P* < 1.139e-11				

Finally, we also conducted a post-hoc whole-brain random effects analysis (see Supplementary data). Briefly, in that analysis, we computed the linear regression model described above, for each voxel in each participant. Analyses were constrained to gray-matter voxels by NSD-provided mask images. A map of *β1* values (regression coefficients on the social interaction rating variable) was computed for each participant. These were transformed to MNI space and submitted to a whole-brain random-effects analysis in SPM12. At a low (uncorrected) voxelwise threshold, we found regions of significant *β1* in and around the regions of interest tested here, as well as in some posterior and anterior superior temporal regions.

#### Results of GAM models

Overall, the results from the GAM analyses were similar to the linear models (see Tables 5–7). In the EBA, there was a significant contribution of social interaction to brain responses in the left but not the right EBA; this difference was significant, *t*(171) = 3.99, *p* = 9.64e-05. Social interaction was related to variance in the FFA, but not FBA. As in the linear models, for all body-selective regions, but not for the FFA, there was a significant contribution from the subjective measure of animatedness in the images. In all regions, the proportion of surface area containing people, and the number of individual people, were significantly related to brain activity. In the left EBA, we found a significant influence of the spatial distribution of people, favouring images with a biased distribution of people in the contralateral side of space, in line with the marginally significant effect found in this region in the linear model.

**Table 5.** Summary of the generalized additive model of extrastriate body area (EBA) responses.

Left EBA	Estimate	SE	*t*	*P*
(Intercept)	553.74	9.89	56.00	<2e-16
	edf	Ref.df	*F*	*P*
Social interaction	1.00	1.00	7.99	0.005262
Animatedness	1.30	1.54	41.95	<2E-16
Manual caption	1.00	1.00	0.21	0.651525
Number of people	2.06	2.39	6.80	0.000796
Left–right distribution	1.00	1.00	2.79	0.096477
Surface area	3.26	3.88	17.09	<2E-16
R-sq.(adj) = .63 Deviance explained = 65%				
REML = 1117.6 Scale est. = 17 794 *n* = 182				

Right EBA	Estimate	SE	*t*	*P*
(Intercept)	557.7	11.6	48.09	<2e-16
	edf	Ref.df	*F*	*P*
Social interaction	1.00	1.00	2.34	0.127862
Animatedness	1.06	1.13	71.46	<2E-16
Manual caption	1.57	1.96	0.46	0.644414
Number of people	1.91	2.24	8.59	0.000186
Left–right distribution	1.00	1.00	1.65	0.200379
Surface area	3.35	3.97	20.64	<2E-16
R-sq.(adj) = .613 Deviance explained = 63.4%				
REML = 1145.7 Scale est. = 24 485 *n* = 182				

Abbreviations: REML = restricted maximum likelihood; edf = estimated degrees of freedom; REF.df = reference degrees of freedom; R-sq.(adj) = adjusted R-squared.

**Table 6.** Summary of the generalized additive model of FBA responses.

Left FBA	Estimate	SE	*t*	*P*
(Intercept)	501.07	10.67	46.95	<2e-16
	edf	Ref.df	*F*	*P*
Social interaction	1.00	1.00	3.46	0.0645
Animatedness	2.05	2.55	24.28	<2E-16
Manual caption	1.00	1.00	0.08	0.7847
Number of people	1.93	2.25	10.46	3.39E-05
Left–right distribution	1.00	1.00	0.04	0.8381
Surface area	2.85	3.43	15.54	<2E-16
R-sq.(adj) = .542 Deviance explained = 56.6%				
REML = 1130.7 Scale est. = 20 729 *n* = 182				

Right FBA	Estimate	SE	*t*	*P*
(Intercept)	651.11	12.13	53.66	<2e-16
	edf	Ref.df	F	p
Social interaction	1.00	1.00	0.81	0.368183
Animatedness	1.63	2.02	20.15	<2E-16
Manual caption	1.00	1.00	0.01	0.923132
Number of people	1.90	2.22	8.66	0.000166
Left–right distribution	1.00	1.00	0.00	0.957103
Surface area	3.29	3.91	17.50	<2E-16
R-sq.(adj) = .515 Deviance explained = 54.1%				
REML = 1153.5 Scale est. = 26 798 *n* = 182				

Abbreviations: REML = restricted maximum likelihood; edf = estimated degrees of freedom; REF.df = reference degrees of freedom; R-sq.(adj) = adjusted R-squared.

**Table 7.** Summary of the generalized additive model of FFA responses.

Left FFA	Estimate	SE	*t*	*P*
(Intercept)	572.37	10.38	55.13	<2e-16
	edf	Ref.df	*F*	*P*
Social interaction	1	1.001	5.137	0.0247
Animatedness	1.001	1.001	1.034	0.3108
Manual caption	1.907	2.402	0.914	0.5016
Number of people	2.065	2.395	15.047	4.32E-07
Left–right distribution	1.001	1.001	0.018	0.8939
Surface area	2.985	3.573	33.162	<2E-16
R-sq.(adj) = .593 Deviance explained = 61.5%				
REML = 1126.1 Scale est. = 19 616 *n* = 182				

Right FFA	Estimate	SE	*t*	*P*
(Intercept)	655.32	10.93	59.98	<2e-16
	edf	Ref.df	*F*	*P*
Social interaction	1	1	6.142	0.0142
Animatedness	1	1.001	0.552	0.4586
Manual caption	2.351	2.949	2.016	0.1322
Number of people	3.163	3.572	10.677	1.38E-06
Left–right distribution	1	1.001	0.941	0.3335
Surface area	3.928	4.588	34.165	<2E-16
R-sq.(adj) = .663 Deviance explained = 68.6%				
REML = 1139.7 Scale est. = 21 729 *n* = 182				

Abbreviations: REML = restricted maximum likelihood; edf = estimated degrees of freedom; REF.df = reference degrees of freedom; R-sq.(adj) = adjusted R-squared.

**Figure 7.**
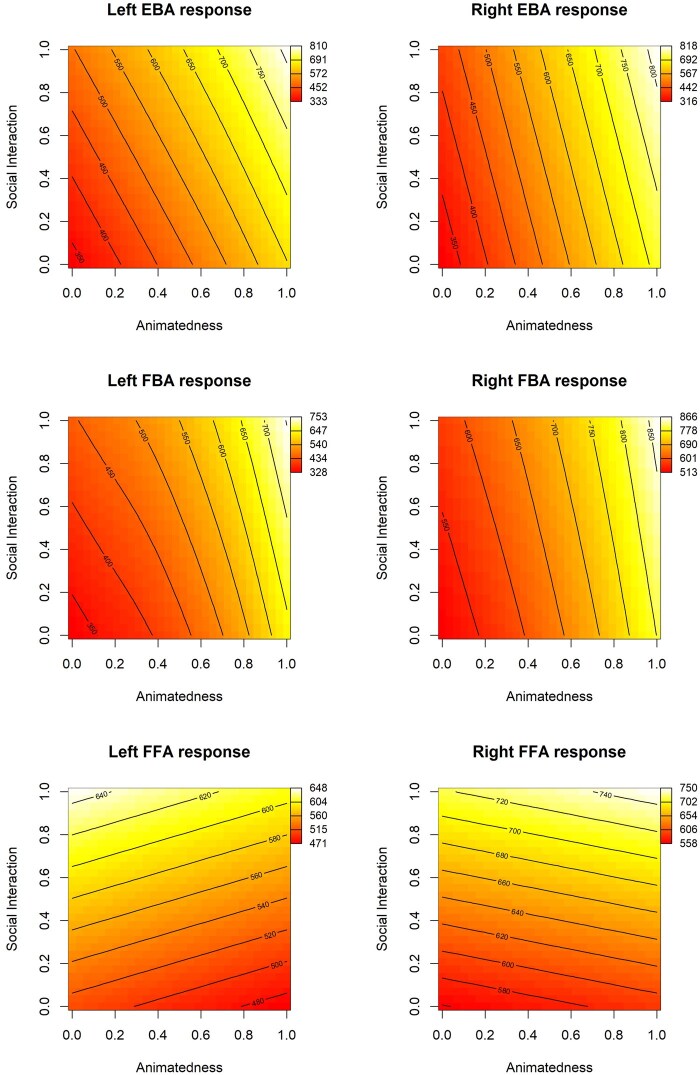
Heat map plots of the fitted relationships between social interaction ratings of images, the subjectively rated ‘animatedness’ of the actors in the images, and the mean activity in key brain regions of interest. Other conventions as in Fig. 5. Note the sensitivity to animatedness shown in body selective EBA and FBA is not mirrored in face-selective FFA.

**Figure 8.**
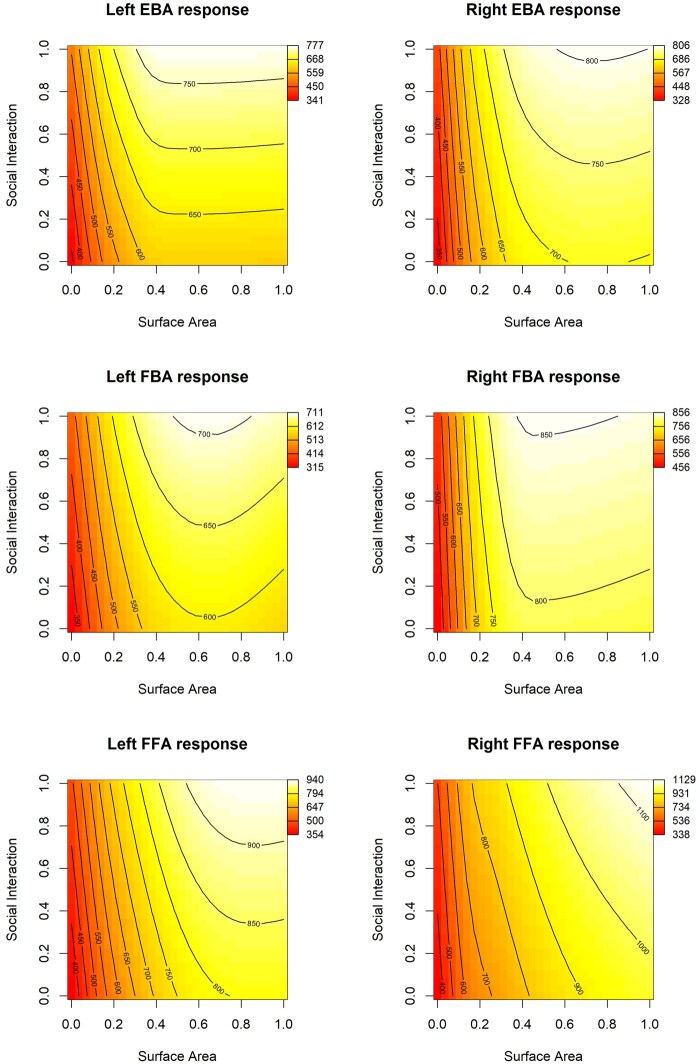
Heat map plots of the fitted relationships between social interaction ratings of images, the objectively measured proportion of the image surface area occupied by people in the images, and the mean activity in key brain regions of interest. Other conventions as in Fig. 5. Note all regions are highly sensitive to the overall surface area occupied by persons, in addition to sensitivity to social interactions.

**Figure 9.**
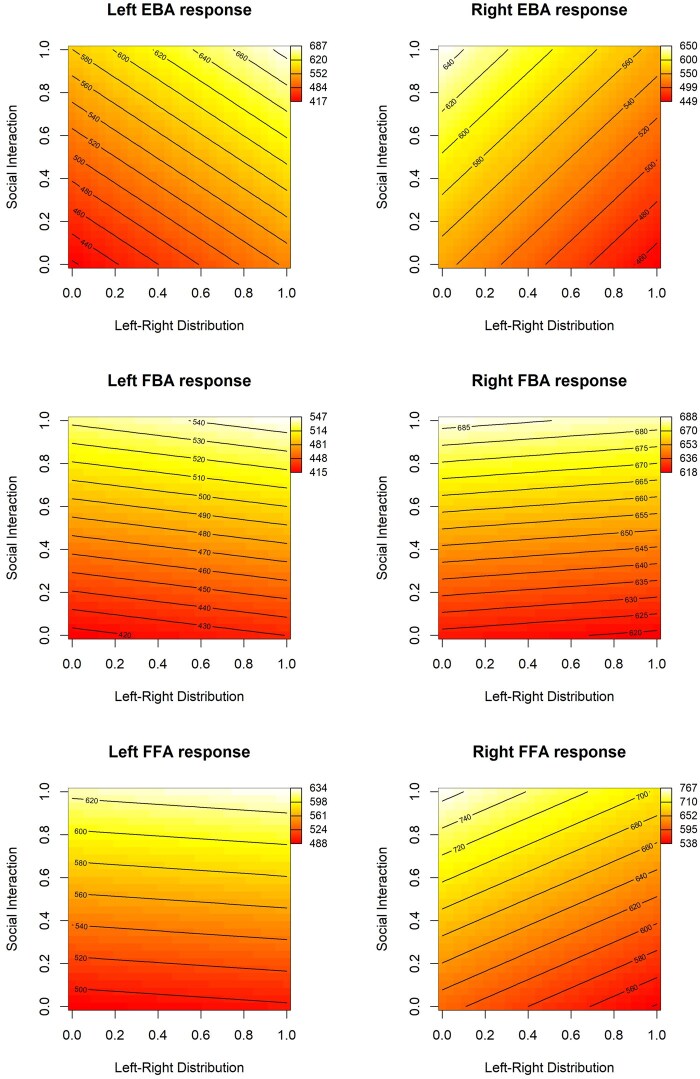
Heat map plots of the fitted relationships between social interaction ratings of images, the objectively measured left–right distribution of the actors in the images, and the mean activity in key brain regions of interest. The left–right distribution index approaches zero to the extent that people are depicted entirely on the left of the image, and one to the extent they are depicted on the right of the image. Other conventions as in Fig. 5. Note the contralateral gradient of sensitivity to people in the images in EBA, in contrast to weaker or absent contralateral biases in the other regions of interest.

Finally, the generalized additive models offer a way to visualize the relationships between predictor variables and brain activity in our ROIs. In Figs. 6–9, we illustrate the relationship between social interaction ratings and each of four other predictor variables. As an aid to interpreting the activation magnitudes shown in these heat maps, note that Fig. 3 shows the distribution of response magnitudes in each region.

## Discussion

We found a significant positive influence of social interactions in static images on the mean activity of occipitotemporal ‘social vision’ regions that were defined on the basis of their selective response to individual bodies or faces. For this finding to be interpretable, of course, we needed to exclude the possible confounding contributions of other variables. Indeed, our analyses also identified significant relationships between brain activity and both low-level and high-level variables, which were often partially correlated with the judgments of social interaction content in the images.

The proportional surface area of an image that was occupied by people was significantly related (above and beyond the other variables) to increased responses in all regions tested. The number of people depicted also explained a significant proportion of variance in ROI responses, often in a negative direction. This pattern is consistent with previous findings of strong responses to individual faces or bodies in selective regions, where typically a single person occupies much of an otherwise empty image. In contrast, in an image of a large crowd, the details of individual faces and bodies may be occluded or indistinct. A denser sampling of images with a varying number of depicted individuals could help identify whether the response profiles of face- and body-selective images depend continuously on that variable, or instead there may be a discontinuity at which ‘persons’ become ‘people’ (cf Phillips et al. 2018).

We found a significant positive relationship between the animatedness implied in an image and brain responses in most of the regions of interest tested. An advantage of the multiple regression approach is that this cannot be explained entirely on the basis of surface area, in the case that dynamic postures take up more image area than more compact, passive ones. Likewise, including this measure helps confirm that social interactions are not driving activity solely due to an increase in implied motion that might result, for example, in cooperative or competitive interactions [Similarly, Landsiedel et al. (2022), found that motion ‘per se’ did not account for sensitivity to social interactions in pSTS and EBA]. Finally, this result provides a reality check on the precision by which we localized ROIs. In the body-selective FBA, but not in the closely adjacent face-selective FFA, responses were significantly modulated by animatedness ratings, which would be expected to largely reflect gross body posture rather than the appearance of the face.

A further reality check was provided by the modest modulation of responses in the EBA as a function of spatial position and hemisphere. In line with previous results, we found a trend towards larger responses in each region to the extent that the spatial distribution of people was contralateral (Hemond et al. 2007, Chan et al. 2010, Silson et al. 2022) and indeed some evidence that this bias is greater in the left than the right hemisphere, as shown by Herald et al. (2023). This analysis is limited for two reasons, however. First, the distribution of persons over the images was concentrated towards the centre of the image (see Fig. 4), following typical photography conventions. Second, the participants in Allen et al. (2022) had 3 s to examine each image, during which time they would have re-fixated the image many times. In light of those limitations, it is notable that the contralateral biases of body-­selective regions are still detectable.

The relatively long image duration in Allen et al. (2022), as well as the use of complex images, may also help to explain why we found a broad positive relationship between activity and social interaction across all of the regions we tested. This is in some contrast to previous studies that found more segregated responses. For example, Gandolfo et al. (2024) found circumscribed fMRI responses to facing as opposed to non-facing human dyads around the EBA, complemented by neurostimulation evidence for a particular contribution of the left hemisphere region. In that study, the images were extremely simple (facing or non-facing dyads) and were not presented long enough to allow for extended exploration. In spite of those differences, the present results (especially from the GAM model) also found an enhanced sensitivity to social interaction in the left EBA, relative to the right EBA. Speculatively, the relatively greater sensitivity to laterality in left EBA might be taken to indicate body representations that retain more information about the positions of people in an image, which may in turn be a useful source of cues to the presence of social interaction.

Our results may initially appear to conflict with prior work suggesting that the lateral occipital cortex, including EBA and pSTS, encodes specific visual components that often occur during social interactions (e.g. an action directed towards someone else) rather than being engaged by a more general or abstract concept of ‘socialness’ or ‘interactivity’ (Wurm and Caramazza 2019). In contrast to the present work, that study tested two sets of carefully controlled video stimuli. Thus, the two studies vary along several dimensions, including both stimulus properties (e.g. static vs dynamic) and the degree of stimulus control. Importantly, however, although our models involved ratings of social interactivity rather than specific visual components of social interaction, in our view our results are not inconsistent with Wurm and Caramazza (2019). Social interaction recognition relies on the processing of perceptual features (e.g. facing direction) that predict the presence of a social interaction. While our results suggest that occipitotemporal regions, including EBA, are sensitive to a variety of interactive cues and contribute to the perception of social interaction across contexts, our aim was not to break down the features that contribute to the perception of social interaction. Future studies using controlled stimuli that manipulate these features independently could test the extent to which these separate visual cues to social interaction differentially drive responses in these regions.

Some strengths of the present approach are: (i) using complex, realistic scene images rather than highly posed figures, or abstract geometric stimuli, in contrast with much of the related previous work; (ii) the variable of interest—social interaction—was not highlighted to participants, who instead were performing an orthogonal task; (iii) the high field strength data and advanced modelling applied by Allen et al. (2022) made it possible to measure regional responses at the single trial level; and (iv) the accompanying localizer data made it possible to identify face- and body-selective ROIs with precision at the individual level.

Although the Allen et al. (2022) dataset includes neural responses to a large set of naturalistic images, in practice once we focused on images depicting multiple actors, and those images that were seen by all eight participants, this set was greatly reduced. The small number of participants tested informed our use of a fixed-effect analysis approach, in which item rather than participant was the random variable. For these two reasons the generalizability of our findings may be limited; further studies with larger samples and larger numbers of suitable discrete images could overcome these limitations. The superior temporal sulcus, especially in the right hemisphere has long been known to respond preferentially to human movements (Allison et al. 2000). More recent work demonstrates strong and selective responses to dynamic interactions as well (Isik et al. 2017, Walbrin et al. 2018, Lee Masson and Isik 2021) but rather weak responses to images (Quadflieg et al. 2015, Landsiedel et al. 2022, Puce 2024). Given the evidence that the pSTS is most responsive to dynamic stimuli, and also owing to the lack of a suitable localizer in the Allen et al. (2022) dataset, we did not directly examine the pSTS here. This issue could be a target for future similar studies that include a wide range of movies. Finally, we restricted our analyses to the mass univariate response of each region. There may be richer information about these regions’ properties to be gained from multivariate analyses. Again, to be successful such an approach would likely require a much larger set of multi-person images than found in Allen et al. (2022), given the number of potentially relevant stimulus dimensions spanning low-level and high-level properties.

Conclusion

In sum, with a data-driven approach, we showed how occipitotemporal ‘social brain’ regions are significantly driven by observed social interactions, above and beyond several other potentially confounding variables, and even when such interactions are not task relevant. These findings provide a complement to studies that use experimenter-controlled manipulations of stimulus variables to analyse the specific features of images that elicit a social interaction interpretation and their relationship to brain activity. Both approaches highlight the need to revise the previous focus on how occipitotemporal regions represent single individuals, to include interacting dyads and groups (Papeo 2020). More broadly, this work may help to flesh out proposals about how those regions work together in support of socially relevant behaviours (Yang et al. 2015, Quadflieg and Koldewyn 2017, Pitcher and Ungerleider 2021, McMahon and Isik 2023, Puce 2024).

## Supplementary Material

nsaf057_Supplementary_Data

## Data Availability

The code and data underlying this article are available in the Open Science Foundation repository at https://osf.io/sy6u2/?view_only=12f1c30f14a345df9dc2501acf9ec365. These analyses are based in part on the Natural Scenes Dataset provided by Allen et al. (2022) and found here: https://naturalscenesdataset.org/
