# Supplementary material for: Social brain responses to natural scene images of social interactions
Source: Soc Cogn Affect Neurosci. 2025 Jun 26;20(1):nsaf057. doi: 10.1093/scan/nsaf057 (PMC12342172; doi:10.1093/scan/nsaf057)
Supplement: nsaf057_Supplementary_Data [file nsaf057_supplementary_data.docx]

**Supplementary Materials for “Social brain responses to natural scene images of social interactions” by Ilona Martynenko, Kami Koldewyn, Paul E. Downing**

Early visual cortex analysis

We defined, for each participant, an “early visual cortex” (EVC) region of interest. The Allen et al. (2022) dataset includes a mask for visual streams, derived from visual field mapping scans, which segments a number of broad visual regions for each participant. We selected the “early” stream, encompassing, per Allen et al., the V1, V2, and V3 regions. The results of the linear regression modelling are reported below (**Table S1**), following the same conventions as the linear modelling of other ROIs in the main text. Sample images of the EVC region of interest are shown in **Figure S1**.

| ***EVC*** | Estimate | SE | t | p |
| --- | --- | --- | --- | --- |
| (Intercept) | 538.66 | 27.22 | 19.79 | <2E-16 |
| Social Interaction | **-77.10** | 36.23 | -2.13 | 0.0347 |
| Animatedness | -140.90 | 24.13 | -5.84 | 2-50E-08 |
| Manual Caption | 46.78 | 32.03 | 1.46 | 0.1460 |
| Number of People | 17.15 | 20.76 | 0.83 | 0.4097 |
| Left-Right Distribution | -37.15 | 42.10 | -0.88 | 0.3788 |
| Surface Area | 168.31 | 39.56 | 4.25 | 3.41E-05 |
| Residual standard error: 88.17 on 175 df | | |  |  |
| Multiple R-squared: 0.2993, Adjusted R-squared: 0.2753 | | | |  |
| F(6, 175) = 12.46, p < 1.139e-11 | |  |  |  |

***Table S1.*** *Summary of the linear model results for the analysis of early visual cortex (EVC) responses.*


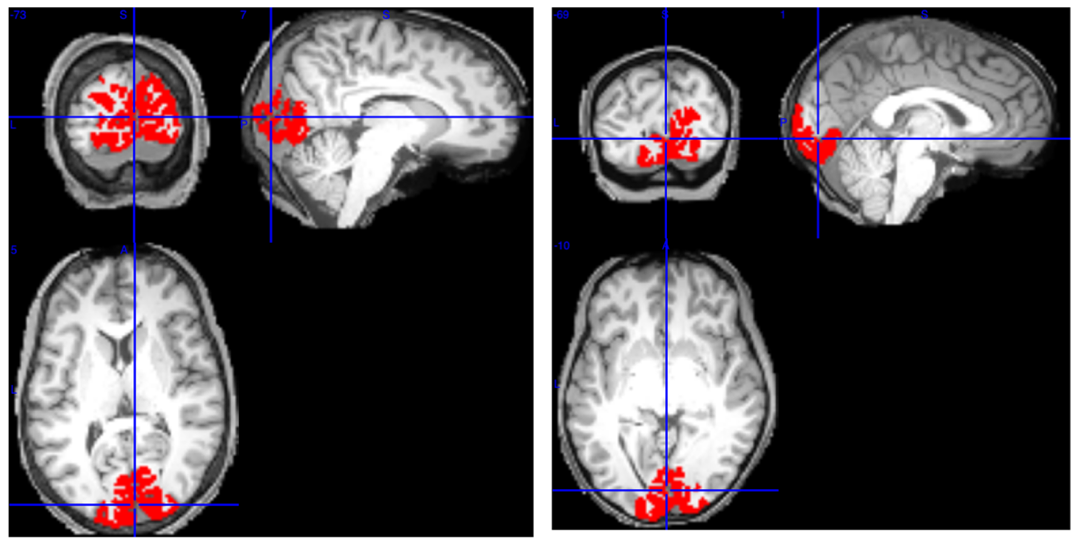


***Figure S1.*** *Illustration of early visual cortex regions of interest from Participants 1 and 2 from Allen et al. (2022).*

Whole-brain analysis

We conducted the same linear regression analysis that we applied to ROIs, for each voxel of the whole brain:

*Brain Response = β0 + β1 ∗ Social Interaction Ratings + β2 ∗ Number Of People + β3 ∗ Left-Right Distribution + β4 ∗ Manual Caption + β5 ∗ Animatedness Ratings + β6 ∗ Surface area + ε*

This was performed in individual participants in native space, restricted to a per-participant mask including only grey-matter voxels. The measure of interest for each voxel was β1: the extent to which the response to the selected images was explained by social interaction ratings, above and beyond the contribution of the other variables. This results in a “beta map” per participant. These maps were transformed into MNI space using tools and transformation matrices provided as part of the Natural Scenes Dataset, and a second-level model applied in SPM12, comparing betas against zero in a one-tailed t-test. With 8 participants, this study is not well-powered for random-effects tests so we set a low threshold (p < 0.01 uncorrected) to limit Type II errors. As seen in Figure S2, the primary regions for which social interactivity shows predictive power at the whole-brain level are in the lateral occipito-temporal cortex, in and around the typical location of EBA and posterior STS, and also in the fusiform gyrus, around the typical locations of the FBA and FFA. While this post-hoc analysis does not permit strong conclusions, it does indicate that the ROI analyses presented in the main text do not miss out large additional key regions in which social interaction in natural images may predict brain responses.

***
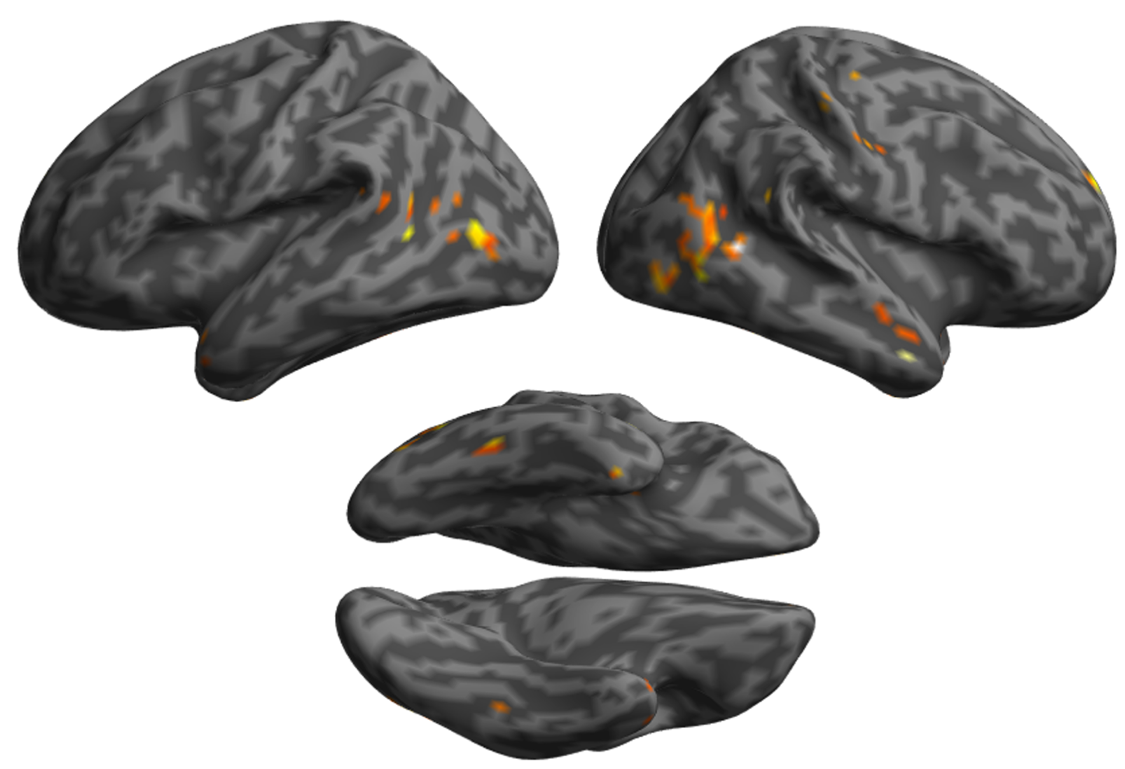
***

***Figure S2.*** *Results of a post-hoc, whole-brain random effects analysis of the linear contribution of “social interaction” judgments to responses to selected images from Allen et al. (2022). Note due to low power a low threshold (p < 0.01 uncorrected) has been applied.*
